# Supplementary material for: Are carbon emissions trading and green financial instruments synergistic? -Comprehensive quantitative research based on content analysis
Source: PLoS One. 2024 Mar 7;19(3):e0298601. doi: 10.1371/journal.pone.0298601 (PMC10919601; doi:10.1371/journal.pone.0298601)
Supplement: S1 Data — (ZIP) [file pone.0298601.s001.zip › Data/地级市Z_F工作报告.zip!/║■▒▒/╛ú╓▌╩╨/└┤╫╘╣1⁄2╓┌║┼í╛┬φ┐╦╩2╛▌═°í┐_2012.docx]

现在，我代表市人民政府向大会作工作报告，请予审议，并请各位政协委员和列席人员提出意见。

2011年，新一届市政府面对复杂严峻的国内外经济形势和旱涝急转等自然灾害，在省委、省政府和市委的正确领导下，在市人大、市政协的监督支持下，团结带领全市人民，把握大势，沉着应对，主动作为，统筹做好保增长、优结构、控物价、惠民生等各项工作，全市经济社会发展呈现三个历史性的重大变化：一是经济总量迈上新台阶。地区生产总值过千亿元、增长13.4%，标志着荆州经济昂首跨入“千亿元俱乐部”。二是战略层面提升新区位。省委、省政府作出实施“壮腰工程”战略部署，标志着荆州发展步入加快振兴的新阶段。三是开放开发呈现新态势。成功获批国家级承接产业转移示范区，荆州开发区晋升国家级开发区，标志着荆州开放竞争优势显著增强。我们可以自豪地说，“十二五”开局之年，实现了良好开局。

农业特色不断彰显。面对三季连旱和秋季汛情，狠抓抗灾复产、结构调整，优质粮棉油、水产、畜禽、瓜果菜、林木等产业板块发展壮大。粮食生产实现“八连增”，监利县连续两年荣获“全国粮食生产先进县标兵”称号。油菜总产1116.4万担，连续三年突破千万担。棉花总产297.2万担，居全省榜首。淡水产品达到108万吨，稳居全国第一。

新农村建设力度加大。新建通村公路548公里，新增农村安全饮水人口32.5万人，植树造林24.3万亩。完成水利投资10.4亿元，新增农田灌溉面积50万亩、除涝面积60万亩，更新改造涵闸泵站15座，除险加固中小型水库15座。仙洪试验区、省级试点镇、市级示范区和县级试点村建设加快推进，农村生产生活条件进一步改善。

工业支撑作用加强。争取上级调度资金3.68亿元、项目补助资金5.97亿元，市本级安排各类企业发展资金6500万元，提供担保贷款25亿元，帮助企业渡难关、扩生产、抓技改。规模以上企业新增94家、达到688家，完成规模以上工业增加值355.2亿元、增长25.1%，连续四年超全省平均增幅。销售收入过10亿元的企业新增4家、达到17家，福娃集团率先突破50亿元，白云边突破30亿元。

发展后劲显著增强。完成固定资产投资773.7亿元、增长37.4%，其中工业投资405.6亿元、增长53.5%。招商引资到位资金320亿元、增长24%，其中工业项目到位资金219亿元，占比达到69%。引进益茂纯电动车、长江镍业、大明水产二期、小天鹅洗衣机二期、中化农药、华油科技、大和重工、宜化磷酸二铵等一批投资10亿元以上的大项目。“洪城”、“洪湖清水”荣获“中国驰名商标”，“洪湖莲子”、“笔架鱼肚”分别荣获“中国地理标志产品”称号和“国家地理标志证明商标”。

城市创建成果丰硕。“四城同创”深入人心，市民素质和城市品位不断提升。荣获全国“双拥”模范城市和省级文明城市称号。府场镇等6个文明村镇和荆州供电公司等7个文明单位受到全国表彰，涌现出王锟、关正世、陈明清等一批先进模范人物。实施园林绿化工程，园林城市创建顺利通过国家验收，松滋市、公安县通过省级园林城市验收。扎实开展“城管行动”，城管检查评比进入全省第一方阵。

现代服务业加快发展。社会消费品零售总额达到556.8亿元、增长17.9%。成功举办全国大遗址保护现场会暨荆州高峰论坛和第二届中国荆州淡水渔业博览会，熊家冢遗址博物馆等大遗址保护工程、“洪湖岸边是家乡”等景区建设进展顺利，洈水风景区纳入鄂西圈核心景区。我市被确定为“中国三国文化之乡”。接待游客1200万人次，实现旅游总收入69亿元、增长32.7%。外贸出口7.7亿美元，增长34.1%。

人民生活水平进一步提高。完成财政总收入74.5亿元，较上年同口径完成数增长25.9%，其中地方一般预算收入44.3亿元、增长32.1%，民生投入占总支出的一半以上。预计城镇居民人均可支配收入16513元、增长12.3%，农民人均纯收入7664元、增长18.8%。金融机构存款余额1226亿元、增长18%，贷款余额548亿元、增长23.2%，连续四年获得“A级金融信用市州”称号，实现“最佳金融县市”全覆盖。物价上涨势头得到遏制，建立起社会救助、保障标准与物价上涨挂钩的联动机制，居民消费价格涨幅低于全国、全省平均水平。新增城镇就业8.5万人、农村转移就业4.9万人，城镇登记失业率控制在4.25%以内。城镇医保、新农合基本实现全覆盖，城乡低保实现动态应保尽保。保障性住房建设年度任务全面完成。

政府自身建设进一步加强。坚持依法行政，自觉接受人大法律监督和政协民主监督，主动加强与各民主党派、工商联、无党派人士、人民团体的联系，认真落实人大常委会决议，办理人大代表建议100件、政协委员提案246件。十件实事全面完成。认真开展“治庸问责”活动，行政效能进一步提升。实行工业项目行政审批全程代办，精减审批项目43个，成为全省行政审批项目最少的市州之一。开展反腐败斗争，政风建设、廉政建设取得新的成效。政务公开、政务督查、新闻发布、应急管理得到加强。

抢抓中部崛起战略机遇，认真研究国家主体功能区规划，积极向上争取政策，主动做好工作对接，我市纳入全国老工业基地调整改造规划，成为国家级承接产业转移示范区、国家“北煤南运”大通道水铁联运重要节点和长江中游区域性综合交通运输枢纽，松滋市纳入全国第三批资源枯竭型城市，荆州发展上升到国家层面的区域战略。省委、省政府出台政策，编制规划，召开现场会，全方位支持荆州加快振兴。“壮腰工程”成为省“两圈一带”战略的重要工作平台，成为荆州发展史上新的里程碑，必将激励我们立足新起点，凝聚新力量，创造新辉煌。

各位代表！越是发展加快的时候，越要保持清醒的头脑。应该看到，我市经济社会发展中还存在诸多矛盾和问题：经济总量不大，速度不快，质量不高，争先进位的压力较大；开放不够，投资不足，大企业、大项目不多，转型发展的压力较大；地方财力有限，民生欠账较多，维护社会稳定的压力较大；少数部门服务不优、创新不够、执行力不强，还存在一些“庸懒散软”现象。落后不是永恒，压力也是动力。我们要忧荆州之发展，树荆州之志气，图荆州之振兴，努力把荆州经济社会发展推向新的高度。

2012年，是实施“十二五”规划承上启下的重要一年，是实施“壮腰工程”打基础、见行动的重要一年。做好今年的工作，关系振兴大局，关系荆州未来。

展望2012年，发展机遇前所未有。国家实施“稳中求进”的宏观政策，加强水利建设、加大粮食主产区利益补偿力度，支持荆州承接产业转移、老工业基地调整改造和大遗址保护片区建设，特别是省委、省政府支持荆州加快振兴，都将为荆州发展带来千载难逢的机遇。我们只有顺势而为，乘势而上，才能让荆州振兴的步伐越走越快。

今年政府工作的总体要求是：认真贯彻落实党的十七大、十七届五中六中全会、中省经济工作会议和市第四次党代会精神，以科学发展观为统领，以改革创新为动力，以改善民生为根本，全面实施“壮腰工程”，积极承接产业转移，稳中求进、好中求快，着力培育大产业、构筑大交通、建设大城市，努力建设湖北长江经济带“钢腰”。

今年政府工作的主要任务是：

着力推进工业提速提质。壮工业的核心是壮支柱产业。实施“千亿和500亿元产业发展计划”，健全完善培育支柱产业的领导体制、工作机制和配套政策，设立产业发展基金和农产品加工专项资金，促进资源、资金、人才、技术等要素向支柱产业汇聚，加快农产品加工、化工、机械制造、轻工建材、纺织服装、高新技术等产业集聚扩张。壮工业的重点是壮龙头企业。实施“百亿元企业培育计划”，在项目用地、资金扶持、金融服务等方面予以倾斜，扶持美的、四机、钢管厂、恒隆、法雷奥、凯乐、沙隆达、楚源、白云边、福娃、大明水产、洪湖浪等一批企业发展成为旗舰型龙头企业。同时，抓大不放小，密切关注中小企业生产经营状况，落实减税政策，及时解决融资难、招工难等问题，促进中小企业快速发展。壮工业的关键是壮新兴产业。舍得以最优惠的政策，扶持战略性新兴产业发展，重点支持高端装备制造、生物医药、新能源、电动汽车等新兴产业发展，鼓励发展循环、绿色、低碳经济，为荆州抢占未来发展制高点打好基础。实施“商标战略”和“质量振兴战略”，加快推进荆州开发区国家级创业中心、省级以上企业工程中心和技术中心建设，鼓励企业开发自主知识产权产品，争创精品名牌。壮工业的基础是壮县域工业。依托资源，突出特色，扶持荆州区石油机械、造纸包装、农副产品加工、光电产业，沙市区机械汽配、针纺服装,江陵县铜铝加工、粮油加工，松滋市酿酒、矿产品加工、精细化工，公安县汽车零部件、塑料新材，石首市化工医药、森工，监利县粮油加工、森工造纸，洪湖市石化装备、水产加工等产业加快发展。积极探索建设用地增减挂钩模式，保障县域重点工业项目用地。

着力推进文化旅游提档升级。围绕建设全国乃至国际知名的文化旅游目的地，充分挖掘现有文化资源，打造旅游精品。加大旅游资源整合力度，推进中心城区旅游资源所有权、经营权、管理权“三权”分离，做实市文化旅游投资平台，做大旅游市场主体。抓好旅游产业与商贸服务业、餐饮业、文化产业有机融合，完善旅游配套服务功能。加大景区景点建设力度，重点抓好荆州古城、楚故都纪南城国家考古遗址公园、荆州文博园和垄上行文化产业园建设，全面启动海子湖生态文化旅游区建设，抓好洪湖至天鹅洲旅游规划景区、松滋洈水风景区等项目建设。力争熊家冢遗址博物馆、“洪湖岸边是家乡”等重要景区在“五一”前后正式开放，并以此为契机开展市场营销，使之成为荆州旅游的新亮点。积极对接高铁，推动旅游深度开发，全方位布局旅游相关产业，借助各种形式的高铁旅游联盟，加强城市合作，开拓客源市场，迎接高铁时代的到来。力争实现旅游收入90亿元，增长30%。扶持新闻传媒、数字动漫、文化创意等文化产业快速成长。

加快建设快速通道。着眼构筑“内畅外联”的快速交通体系，在重点项目推进、体制机制创新、区域一体化上实现重大突破，尽快凸显荆州交通区位优势。加快江南高速、洪监高速、荆监一级公路（郝穴至容城段）建设进度，开工建设荆州至松滋一级公路。抓紧做好沙市至公安高速、武汉城市圈环线洪湖段、318国道荆州段改扩建工程、江北高速、潜江至石首高速前期工作，力争年内开工。完善沪蓉高铁荆州火车站配套设施，力争“五一”前后荆州正式通达客运列车。积极推进北煤南运大通道（荆岳铁路）建设。加强境内国省干线、县乡公路、村级公路建设和危桥改造，努力提升荆州公路网纵深服务能力。

加快建设综合交通枢纽。立足高起点，搭建生产要素流动的平台，释放水、公、铁联运效应，畅通人流、物流、信息流。依托荆州火车站，加快建设郢城综合客运枢纽站。依托长江黄金水道，加快建设盐卡保税物流园、李埠港物流园、松滋车阳河物流中心。依托工业园区，加快建设荆岳综合物流园、襄荆物流中心、荆州开发区货运中心。启动沙市机场迁建前期工作。

精心规划城市。加强全域规划，编制完成城乡总体规划，抓好中心城区、节点城市、沿江沿路城镇带和中心镇、特色镇规划，实现规划全覆盖。加强全景规划，按照城乡规划“一张图”、地上地下基础建设“一张网”、产业布局“一盘棋”的思路，编制各类专项规划、控制性详细规划及修建性详细规划，避免“千城一面”、“千楼一面”。加强全程监管，进一步理顺城乡规划管理事权，严格规划执法，维护规划权威，用规划引领发展，用规划指导建设。

精细管理城市。进一步推进环卫作业市场化，实施市政、园林建管分离，明确分区分段监管责任，推进管理“网格化”。整合公安、电信等信息资源，建立集采集、处理、监管、考核为一体的信息系统，推进管理“数字化”。完善考评制度，进一步强化各级政府城市管理责任，建立健全发现及时、处置快速、解决有效、监督有力的长效机制，推进管理“规范化”。建立对县市半年一检查、城区一月一考评的城管工作制度，鼓励和支持各县市按国家和省级标准积极开展城市创建活动。从细节抓起，从点滴做起，重点整治乱搭乱建、道路拥堵、广告杂乱、垃圾乱扔、沿街为市等顽疾，全面提升城市形象。

积极承接产业转移。国家批准荆州承接产业转移示范区主体为荆州市全境，辐射带动周边地区，并要求建设成为中部地区承接产业转移的优秀示范区、人水和谐可持续发展的先行区、跨区域合作与产业转型发展的综合实验区和湖北经济发展的重要增长极。我们要充分发挥这一“国字号”金字招牌的作用，优化投资结构，促进产业集聚升级。加快拓展承接平台，推进园区规模扩张，力争年内荆州开发区新增园区10平方公里、县市区新增园区5平方公里。加强园区水、电、路等基础设施和商、住、娱等配套建设，增强园区承载能力。支持荆州开发区全方位提升招商引资水平，全方位加强基础设施建设，全方位提升管理服务能力，打造中部地区承接产业转移的核心区、示范区。更加务实、理性招商引资，强化驻点招商、产业链招商、专业化招商，形成招商引资、项目落地、企业服务相互衔接、环环相扣的工作专班和机制，力争全年招商引资到位资金410亿元、增长30%以上。探索产业转移承接机制，发展共建共享的“飞地经济”。

深入推进各项改革。深化财政改革，切实抓好税收征管，提高地方税收占一般预算收入的比重，做大财政蛋糕；加强预算管理和非税收入管理，严控一般性支出，扩大公共财政支出，分好财政蛋糕；健全政府举债机制，防范和化解财政风险。深化金融改革，大力引进村镇银行、股份制银行，培育发展保险市场，规范发展小额贷款和各类担保公司；积极推动农村信用合作社改制，推进银企深度合作，力争金融贷款余额突破670亿元。鼓励支持企业上市融资和上市企业再融资。深化国企改革，加强与央企对接，推进国企产权多元化、资产资本化、资本股份化、股份证券化。深化农村综合改革，推进城中村、城郊村、园中村集体资产产权制度创新，健全农村土地承包经营权流转市场，改革小型水利工程管理体制。深化行政体制改革，理顺市区管理体制，做到权、责、利一致；积极稳妥实施食品药品、工商、质监管理体制改革，加快医药卫生、文化体制改革和事业单位分类改革。深化投融资体制改革，鼓励民间资本进入基础设施建设、公用事业、社会事业等领域。

扩大就业和社会保障。实施积极的就业政策，鼓励发展劳动密集型产业、服务业和小型微型企业，扶持创业带动就业，统筹做好大中专院校毕业生、各类就业困难人员和农村转移劳动力就业；依法扩大各类社会保险覆盖面，基本实现城乡居民养老保险全覆盖。探索农村低保、城市低保、医疗救助、农村五保“一条龙”工作模式，巩固城乡低保动态下的应保尽保。适应社会老龄化趋势，加快建设以居家养老为基础、社区养老为依托、机构养老为支撑的社会养老服务体系，努力让老年人老有所养、晚年幸福。建立和完善以公共租赁住房为主体的住房保障体系。

切实维护公共安全。加强和创新社会管理，构建有控制能力的管理体系，努力建设平安荆州。开展“六五”普法宣传教育，强化法律援助，完善人民调解、行政调解、司法调解与仲裁相衔接的大调解体系。严厉打击涉黑、涉恶、涉毒、“两抢一盗”等违法犯罪行为，增强群众安全感。高度重视群众来信来访工作，畅通群众诉求渠道，搭建政府服务平台，切实维护好群众合理诉求。扎实开展“打非治违”专项整治，认真排查、消除各类隐患，突出整顿客运市场隐形挂靠和校车安全，坚决遏制重特大事故发生。大力整顿规范食品药品生产、流通秩序，让群众买得放心，吃得安心。加强应急平台建设，提高突发事件的预防、处置能力。抓好国防动员、民兵预备役工作，支持驻荆部队建设，提高城市人防综合能力。

三、加强政府自身建设

（一）提神鼓劲想干事。壮腰先壮志气，振兴先振精神。站在新的历史起点，我们要以“壮腰工程”为动员令和总抓手，凝心聚力，汇聚能量，筑牢加快振兴的思想基础，坚定加快振兴的信心决心，调动加快振兴的激情干劲。激励每一个荆州人心系荆州、心忧荆州，与荆州发展荣辱与共；拿出志气、豪气，在振兴荆州中建功立业；拿出血性、韧性，让积蓄已久的能量喷薄而出。目标同向，工作同步，发展同心，努力营造干事创业、合力振兴的浓厚氛围。

（三）勇挑重担真干事。大兴调查研究之风，深入基层，承接地气，多到招商一线、工厂工地、城乡社区、田间地头，掌握一手情况，解决实际问题。大兴真抓实干之风，力戒虚华，务求踏实，沉下心，静下气，在招商引资、项目落地、服务企业和群众上下功夫。积极倡导“三短一简”，开短会、讲短话、发短文、简办事，腾出更多时间和精力用于谋发展、抓改革、惠民生、促和谐。大兴“过硬过细”之风，严格绩效管理，强化行政问责，把每一项政策具体化，每一项工作项目化，每一项责任明细化，严肃查处不作为、慢作为和乱作为等行为。

（五）勇立潮头干实事。要勇立改革发展潮头，做人民群众的引路者和贴心人，做依法行政、遵纪守法的带头人。勤政为民，念好“衣食住行、医教保业”民生“八字经”，把解决民生问题作为扩大内需的着力点和助推器，使广大群众分享发展成果，感受公平正义。依法行政，自觉接受人大法律监督、政协民主监督、媒体舆论监督、社会各界监督，认真执行市委决定、人大决议，办好办实人大代表建议和政协委员提案，支持民主党派、工商联及无党派人士建言献策、参政议政。廉洁从政，认真贯彻落实党风廉政建设“一岗双责”制度，教育广大公务员筑牢思想防线，守住道德底线，不越法纪红线，进一步规范行政执法、土地出让、产权交易、招标投标、政府采购等行为，严厉查处违法违纪案件，努力营造风清气正、干净干事的良好氛围。
